# Evaluation of self-cleaning mechanisms for improving performance of roof-mounted solar PV panels: A comparative study

**DOI:** 10.1371/journal.pone.0309115

**Published:** 2024-10-29

**Authors:** Danish Hameed, Allah Ditta, Muhammad Wasif Bajwa, Sibghat Ullah, M. A. Mujtaba, Yasser Fouad, M. A. Kalam, Manzoore Elahi M. Soudagar

**Affiliations:** 1 Mechanical Engineering Department, University of Engineering & Technology (UET), Taxila, Pakistan; 2 Mechanical Engineering Department, University of Management and Technology Sialkot, Sialkot, Pakistan; 3 Mechanical Engineering Department, Mirpur University of Science and Technology (MUST), Mirpur, AJK, Pakistan; 4 Department of Mechanical Engineering, UET Lahore (New Campus), Lahore, Pakistan; 5 Department of Applied Mechanical Engineering, College of Applied Engineering, Muzahimiyah Branch, King Saud University, Riyadh, Saudi Arabia; 6 School of Civil and Environmental Engineering, FEIT, University of Technology Sydney, Sydney, NSW, Australia; 7 College of Engineering, Lishui University, Lishui, Zhejiang, China; 8 Department of Mechanical Engineering, Graphic Era (Deemed to be University), Dehradun, Uttarakhand, India; Chiang Mai University, THAILAND

## Abstract

Solar panel installation is generally exposed to dust. Therefore, soiling on the surface of the solar panels significantly reduces the effectiveness of solar panels. Accumulation of dust also shortens their lifespan and reduces efficiency by about 15% to 20%. A significant reduction in the efficiency of solar photovoltaic panels has been observed due to inadequate insulation and dust deposition or shading. To harness maximum solar energy from solar panels up to their rated capacity, they need to be cleaned periodically. Therefore, the current study focuses on the comparative performance analysis of two distinct types of self-cleaning mechanisms, namely self-cleaning wiper (SCW) and nano-coating method. These methods are economical and sustainable for the standard atmospheric conditions of Pakistan. Solar panels (reference, nano-coated, and self-cleaning wiper mechanism) were placed on the roof of the Mechanical Engineering Department MUST Mirpur AJK for five weeks. Solar irradiance, dust density & performance parameters of these three panels were recorded on weekly basis. It was observed that an increase in the rate of dust deposition negatively affects the conversion efficiency of solar panels. When dust density was increased from 7.5 to 18.15 (g/m^2^), the percentile drops in rated power (50W) for reference, nano-coated, and self-cleaning wiper mechanisms are 37%, 33% and 23%, respectively. Moreover, the payback period of nano-coated and SCW is 1.07 years and 2.79 years, respectively.

## 1. Introduction

Pakistan is a growing nation with insufficient electricity generation resources to fulfill its demands. Power generation reached 24,900 MW in 2022, leaving a gap of 6439 MW [1]. Mainly, conventional energy sources are utilized to serve this purpose, leading to increased carbon footprints. However, with ample solar irradiation averaging at 5.5 kWh/m^2^, solar energy is one of Pakistan’s most fundamental, unpolluted, and untapped renewable energy sources [2]. Photovoltaic (PV) panels have a wide range of applications. They are commonly used to generate electricity in off-grid locations, power remote telecommunications equipment, and provide energy for water pumping and heating/cooling systems [3]. In recent years, there has been a growing interest in using PV panels in water pumping and heat pump applications due to their ability to provide clean and reliable energy with minimal operating costs and maintenance [4]. Various techniques have been developed to boost performance, including adjusting the tilt and orientation of the PV panels, utilizing tracking systems, and implementing energy storage during times of low solar irradiance. To maximize the overall performance of solar panel, the finned paraffin containers with nanofluid flow, a cylindrical reflector, a thermoelectric generator layer, and a silver-water nanofluid spectral filter are integrated. The nanofluid filter exploits tunable optical properties and enhanced heat transfer to boost light absorption and cooling. The cylindrical reflector and thermoelectric layer further augment electricity generation and heat recovery [5–7]. Likewise, in heat pump applications, PV panels can power the system’s components, which results in a highly efficient conversion of solar energy for space heating and hot water. To improve the efficiency of PV-powered heat pumps, high-efficiency components, system design optimization, and energy storage systems are recommended to maximize solar energy utilization [8]. The cross-sectional design for the heat transfer tube in solar systems using tube geometry with copper fins and a graphene nanoplatelet-water nanofluid to enhance overall performance of solar panels [9, 10].

The future and health of humans are in danger due to the fast rise of carbon emissions [11]. With the aid of renewable energy sources, energy demand is satisfied by the use of clean energy [8, 12]. Because of their enhanced efficiency and steadily falling erection and maintenance costs, solar systems are among the most popular renewable energy sources [13]. The solar panel’s temperature, tilt angle, humidity, and dust are all factors that affect the operation of the PV module in addition to its lifespan. The effectiveness of solar panels is impacted by dust, shortening their lifespan and reducing efficiency about 15% to 20%. However, several important problems arise after the erection of solar energy systems, including shadowing, dust and other forms of comparable dirt brought on by various factors, which significantly reduce the energy produced by solar energy systems [14]. Solar PV cleaning techniques and methods are crucial for maintaining optimal performance and efficiency of photovoltaic systems. Recent studies have explored various approaches to mitigate dust accumulation and enhance panel output. Waterless cleaning techniques involve the use of dry methods such as brushes, air blowers, or vacuum systems to remove dirt from solar panels [15]. Dust deposition on solar photovoltaic panels has been a keen interest of many researchers worldwide since 1942 because it significantly impacts PV panel’s performance. Many research has been published in literature to address dirt and dust problems in PV arrays [14, 16, 17]. The 17 different forms of dust can be classified by Darwish et al. [18]. Only six types of dust particles–ash, red soil, limestone, calcium carbonate, sand and silica are crucial for PV panels. Siddiqui et al. [19] showed a relationship between dust buildup and effectiveness in 2011. It suggests a light coating of dust on the surfaces protecting silicon photovoltaic modules does not significantly hinder the passage of sunlight into the inner silicon components.

Solar PV panels are currently cleaned using various techniques, including the traditional brush approach, compressed air dust removal, coating techniques, and robotic cleaning systems. Because there are so many dry regions in Africa and the Middle East, PV module surfaces in solar energy farms are quite dusty [20]. Techniques similar to super-hydrophobic coating have been suggested to stop PV panels from losing efficiency due to the accumulation of both inorganic and organic dirt. Numerous benefits, including anti-graffiti and anti-corrosion, come with the super-hydrophobic thin layer on the surface for self-cleaning PV modules [21]. The electrostatic cleaning system is an additional method that removes sand from solar panels by single-phase high voltage. It has been noted that the suggested cleaning technique decreases PV panel surface dust by 90%. The proposed technology offers several benefits, including low cost with no consumable required for solar power parks in desert areas at minimal latitudes [22]. In the literature on robot cleaning procedures, there are dry cleaning and aqueous cleaning options available [23]. Due to the lack of water in desert areas, a dry-cleaning technique is given that doesn’t require any liquid to clean PV panels. The dry-cleaning device sprays air jet to clean the panel [24]. A new kind of brush has been designed to clean the module, which is inexpensive and doesn’t harm the surface of the PV module [25]. The water-free, automated cleaning system was created domestically to keep PV panels free from dust. An experimental study analysis concluded that the quantity of energy received from PV panels increased by around 6.31% by using a water-free cleaning mechanism over a month [26]. The solar PV water pumping system is intended to operate more effectively using a cleaning mechanism that sprinkles water over the PV plates. By using a specially built water-spraying cleaning mechanism to lower the panel temperature, the system’s efficiency has been dramatically increased [27]. Table 1 summarizes the literature on various solar PV panel cleaning techniques.

**Table 1:** The literature review on various cleaning techniques of solar PV panels.

Ref. No.	Proposed method	Findings	Efficiency, Power and Transmittance losses improvement
[28]	anti-soiling coating (nanoparticles of metal oxide and hybrid binder)	Six polycrystalline PV panels from the same manufacturer and with the same features. Three of them had coatings, and the other three remained uncoated. All the panels were angled southward at a 21° angle.	PV panels produced 5.3 Wh/Wp and 5.2 Wh/Wp of electricity for coated and uncoated, respectively. PV panels had transmittance losses of 12% for uncoated and 10% for coated ones.
[29]	Mechanical removal, Natural removal, Nano-film coatings, Electrostatic removal and Self-cleaning mechanism.	Reviewed methods of removing dust from solar arrays. The region with little rainfall was not suitable for nano film coatings. The surface of the panel could be harmed by mechanical dust cleaning. An electric curtain was the most effective way to remove dust.	No data are available.
[30]	Auto cleaning robot	Built and studied the design of an auto-cleaning robot for PV panels. It is concluded that auto robots were economically profitable.	No data are available.
[31]	Anti-soiling coating	Analyze the anti-soiling coating effectiveness for a 10 MW PV power plant simulation. Three various circumstances, such as wet, dry, and reference, were shown to the glass plates. Economically, it was determined that all three scenarios would result in a 3% profit.	The transmittance losses were enhanced by 11.3% by artificial dusting, while the power output was improved by 3%.
[32]	Dry cleaning is done using nylon brushes and iron glasses.	Transmittance was recovered 90.67% by dry cleaning and 92.7%. By wet cleaning. And does not cause any damage to solar panels.	As a result of the 7% reduction in absolute transmittance, the system’s efficiency dropped by 7%.
[33]	Anti-soiling coating	Sixteen low-standard glass panels from a PV module manufacturer made up the setup in Norway. Only eight of them had a 45° tilted and anti-soiling film coating.	For 10 mg/m^2^ of dust, transmittance was decreased by 0.09% for uncoated glass plates and by 0.11% for coated ones.
[34]	Anti-soiling and anti-reflective coating, Cleaning with water and brushes.	On the top of a seven-story building, there were already three PV arrays, A, B, and C. The PV modules were of the Cu (ln, GA) Se2 type. Array A was left untreated, Array B was cleaned with water and a brush, and C was cleaned with water and a brush and also had an anti-reflective and anti-soiling coating.	The coated array’s power output exceeded the untreated one by 3.6%, and the average transmittance was 3.6% higher for the coated array.
[35]	The cleaning method not described.	The study utilized two polycrystalline PV modules from the same vendor. Each day, one of them was cleaned. The results showed that the bottom of the panel had a significant level of dust accumulation.	Efficiency was found to be decreased by 26.9% compared to the clean solar panel.

The current study focused on designing and developing two self-cleaning mechanisms for removing dust particles from solar PV panels. To serve this purpose, an experimental test rig is installed on the roof of the Mechanical Engineering Department (MED) at Mirpur University of Science and Technology (MUST) in Mirpur, Azad Jammu and Kashmir (AJ&K), Pakistan. This location was chosen for installing the PV panels because heavy traffic passes nearby this building and is in the airborne dust’s path. Moreover, on average, the solar radiation on the tilted surface at this location is more than 800 W/m^2^. Furthermore, cleaning mechanisms were compared based on key performance metrics such as power output, short circuit current, and open circuit voltage. The previous studies have evaluated various water-based, robotic, and coating cleaning approaches [15–25], limitations remain around potential surface damage, water dependency, and long-term maintenance. To address these gaps, the current work proposes two novel self-cleaning mechanisms ‐ a self-cleaning wiper system and TiO_2_ nano-coating technique ‐ and conducts the first direct comparative performance analysis of these original solutions.

## 2. Materials and methods

Maintaining the efficiency of solar energy panels requires routine cleaning, and mostly pure water is utilized to serve the purpose. Soiling decreases the efficiency of the module up to 30%. Three different 50W PV panels were used in the experimental setup: reference panel, nanocoated panel, and self-cleaning wiper panel, which were dusted in the same environment. Two methods that deal with the impact of dirt have been designed and mounted on PV modules; the performance of these modules (SCW mechanism and nanocoated) was compared to the performance of a solar module without a cleaning system, termed as the reference panel. The surface of the panel with the SCW mechanism is cleaned with water, other is coated with nanocoating spray and the values of open-circuit voltage (V_oc_) and short-circuit current (I_sc_) are measured simultaneously. Experiments were performed over a period of five weeks to determine the performance parameters of each solar panel, which were evaluated by using a solar PV analyzer (Prova 200A). The equipment with their specification used in the experimental setup is presented in Table 2.

**Table 2:** Component used for experimental setup.

Electrical Components	Mechanical Component	Chemical Component
Solar Panels	Frame for support	Nano- Coating
DC motor (16W)	Lead screw	
Battery and Pump (12 V)	Wiper	
Light Dependent Resistor	Sliding railing	
Resistors (10k, 4k7, 1k, 390R)		
Transistors (C945 and A1015)		
Digital Temperature Sensors		
Limit Switches		
Relay (12 V)		
Prova 200A		

### 2.1. Solar PV panel

**Fig 1:**
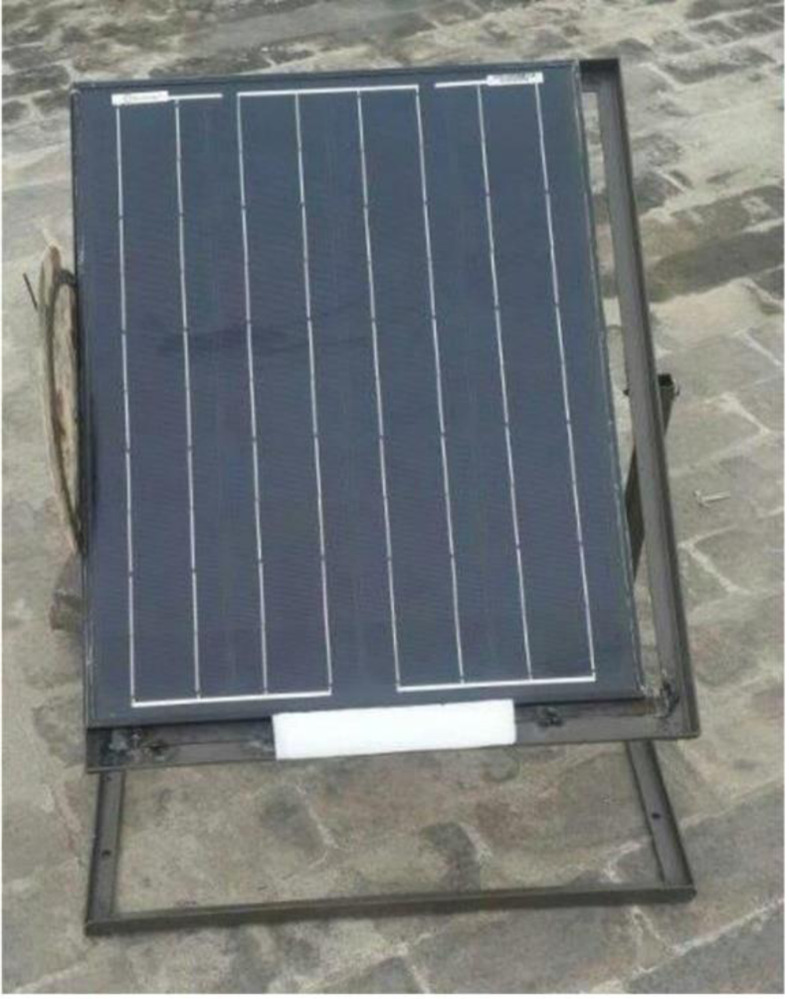
Reference solar PV panel.

Solar cells stacked in series and parallel make up solar PV panels. The relationship between solar cell voltage and current delivered to the load determines the Current-Voltage (I-V) and Power-Voltage (P-V) characteristics of the cell. These characteristics determine the circumstances in which the power gathered from the panel should reach its maximum value. The performance of the PV module utilized for the trial investigation is assessed in this study using the sun irradiance, dust density & performance parameters (I-V, P-V curves and P_max_) weekly. The specifications of the PV module employed for the trial investigation are also shown in Table 3. Three solar panels are used in this study, although only one (the reference panel, depicted in Fig 1) has no cleaning mechanism. The reference panel’s performance is compared to the SCW mechanism panel and the nanocoated panel.

**Table 3:** The electrical specifications of the solar PV panel.

Characteristics	Values
Type	Mono-crystalline
Manufacturer	Suntop, Jiaritong
Voltage, Current	17.5V, 2.85A
Power	50W

#### 2.1.1. Self-cleaning wiper mechanism

A 50W monocrystalline solar panel is mounted on a frame at a 33° angle to get the most sunlight. The inclination angle of a solar panel significantly impacts its efficiency in capturing solar energy. Considering the geographical latitude of Mirpur city, the optimal tilt angle of solar PV panels is adjusted at 33°. The fundamental and essential element that supported the panels and could be adjusted was the supporting frame for the panel, which was ready to accommodate the SCW (self-cleaning wiper) mechanism. Considering the torque requirement to drive the wiper along the rail, a DC motor with a torque of 100 g-cm was selected. The power rating of 16W is adequate considering the intermittent operation of 30 secs every hour. The DC motor was added to supply power, and a railing was put on either side of the panel to assist the wiper in cleaning. The lead screw, which transforms rotational action into linear motion, was attached to the wiper. After that, the water jet powered by a 12V DC water pump was adjusted. A flexible plastic tube of 6mm diameter was used. The tube was fixed on the top of the panel. The pump was rated to deliver 0.5 litres/min of water at a working pressure of 1 bar. During the 45-second cleaning cycle, approximately 250–300 ml of water was required to flush away loose dust particles. A nearby water tank provided the water supply.

**Fig 2:**
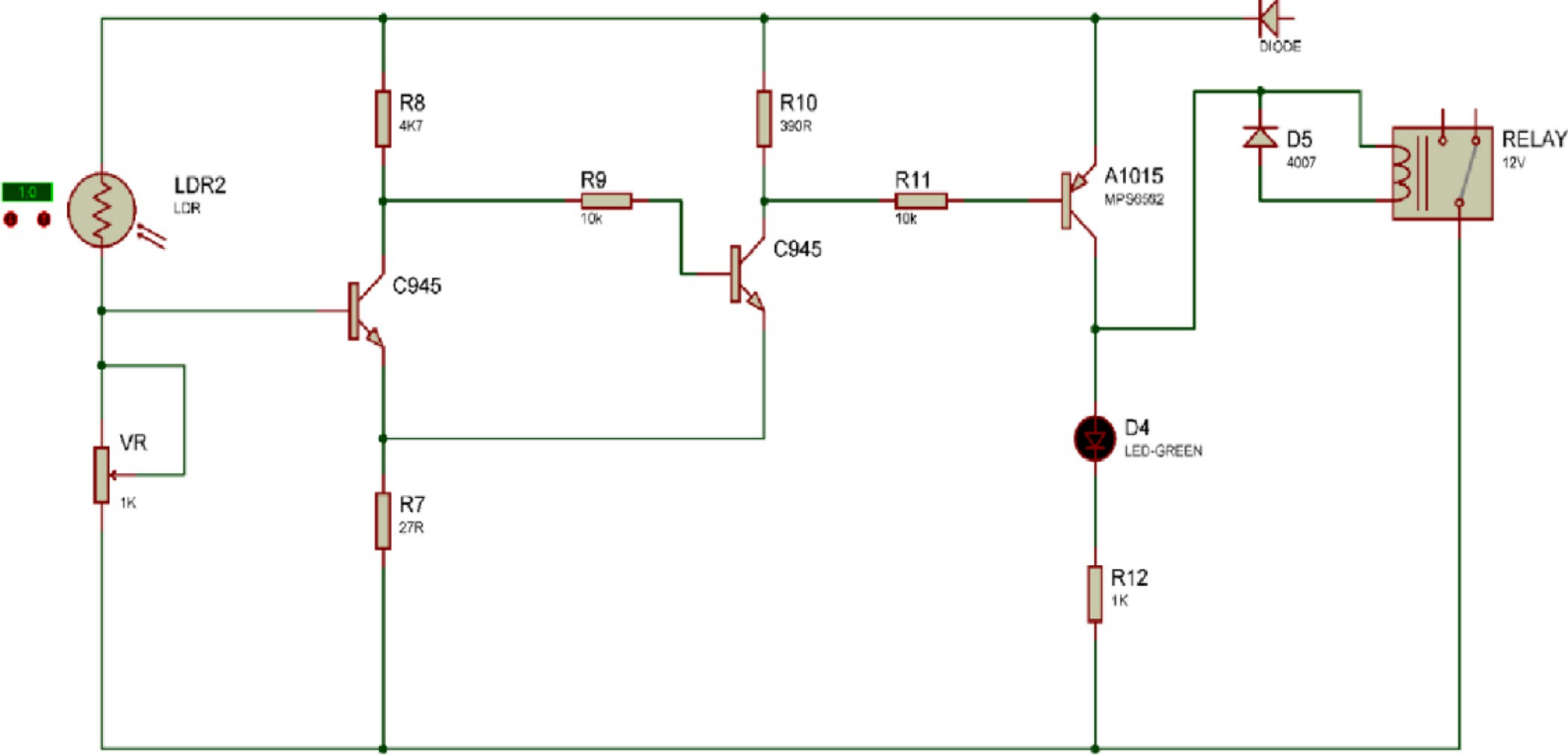
Circuit diagram of SCW mechanism.

The difficult next step was to simulate the electrical circuit of the fundamental design after it had been developed. Different electronic parts that are used to operate the cleaning mechanism, as shown in the H-bridge circuit diagram in Fig 2. The resistance in series on the circuit board regulates the motor’s rotational speed (rpm). A LDR (Light Dependent Resistor) is installed on the PCB; when switches are plugged into the battery terminals, the LDR signals the motor to turn on. The pumping action starts simultaneously, spraying water onto the solar panel. With the aid of a lead screw, the wiper descends as the motor begins to rotate counterclockwise. This procedure starts when sunlight strikes on the LDR and resumes when there is no light.

**Fig 3:**
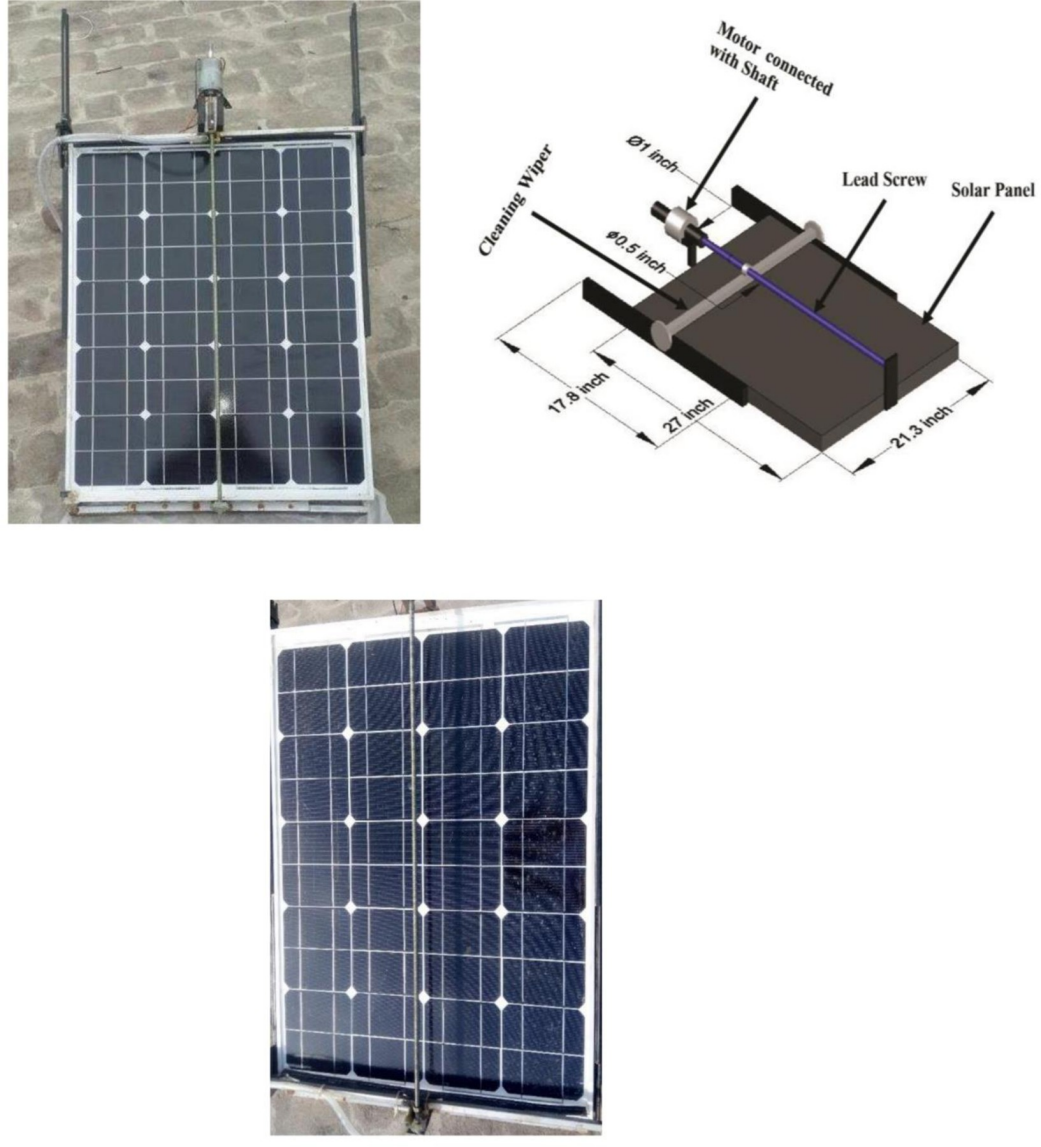
(i) Self-cleaning wiper mechanism, (ii) Schematic diagram of SCW, (iii) Dusted panel.

At each side of the solar panel, a limit switch was positioned at the bottom and the top. When a wiper approaches a limit switch, the limit switch opens the circuit, halts the motor’s action, and the wiper stops at the bottom. When the LDR detects no light, it informs the motor, which turns clockwise and drives the wiper higher until it contacts the limit switch. The self-cleaning mechanism operates for 32 seconds. The experimental setup and schematic diagram of the SCW Panel are shown in Fig 3.

#### 2.1.2. Nano-coated

**Fig 4:**
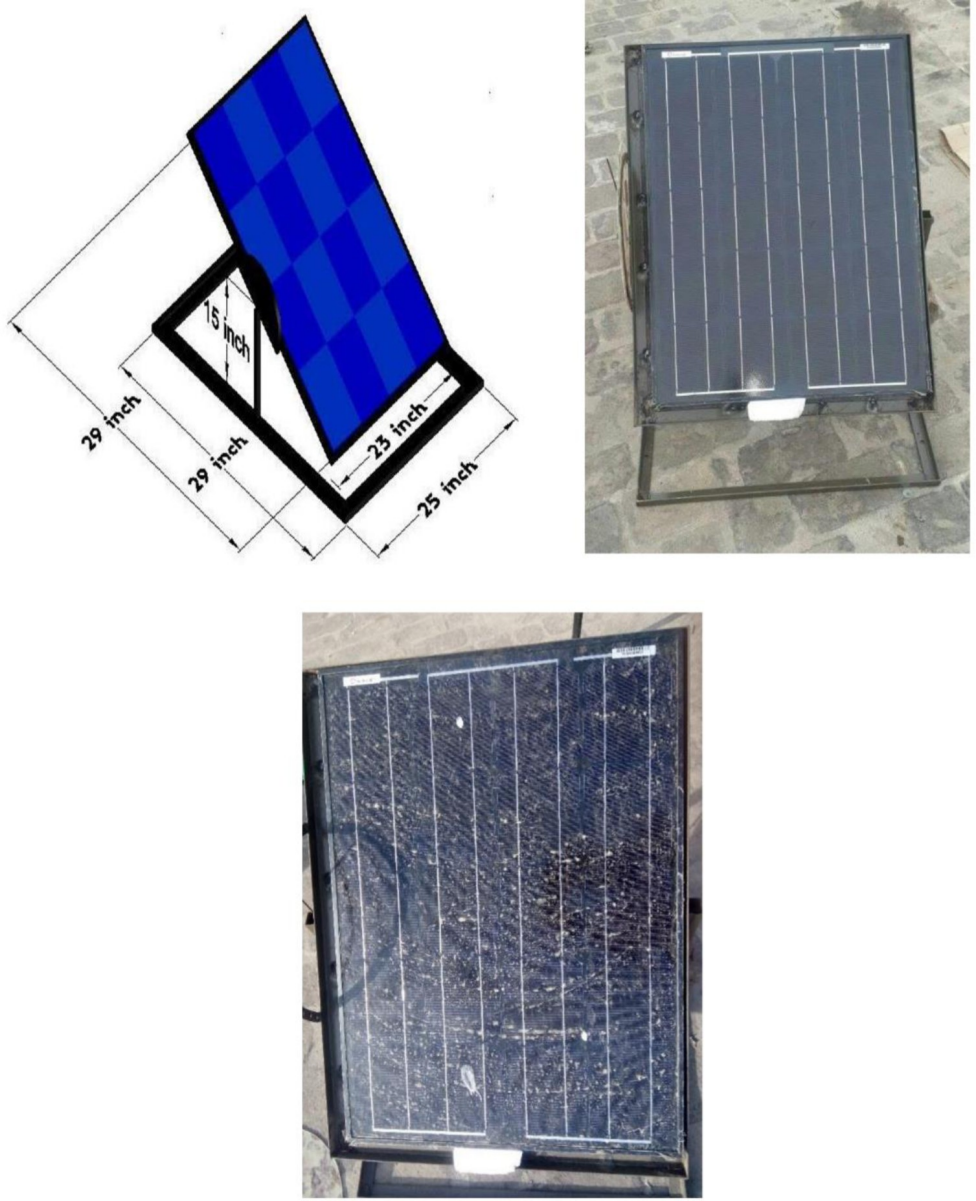
(i) Schematic diagram of the nanocoated panel, (ii) Nanocoated panel, (iii) Dusted panel.

The second mechanism was developed by using nano-coating on the solar panel’s surface. The nano-coating spray used contained TiO_2_ Nanoparticles. A concentration type of impregnation known as nano-coating for solar panels creates a clear covering that shields the surfaces from particles such as dust, oil, and other particulates. A nano-coating spray is sprayed onto the PV panel’s surface using a microfiber cloth. Nano-coated solar panels make the surfaces they’re applied on self-clean. The hydrophilic characteristics of the nano-coating provide a 95% reduction in dust and dirt adhesion. Moreover, the photocatalytic activity of TiO₂ helps in breaking down organic materials and pollutants that accumulate on the surface of the solar panels. TiO₂ coatings are resistant to chemical corrosion and environmental degradation, ensuring that the protective and functional properties of the coating remain effective over a long period. The schematic and experimental setup of the nanocoated PV panel is shown in Fig 4.

### 2.2. Solar PV analyzer

Open circuit voltage, short circuit current, maximum current and voltage at Pmax, and the solar radiation intensity reflected on the solar PV panel were all measured using Prova 200A, a solar PV analyzer [36]. The ambient temperature of PV modules was measured with the help of a digital temperature sensor, and data was stored in an analyzer after experimentation. The output data values are recorded in the PV analyzer with the software in the computer as an Excel file.

### 2.3. Dust measurement

**Fig 5:**
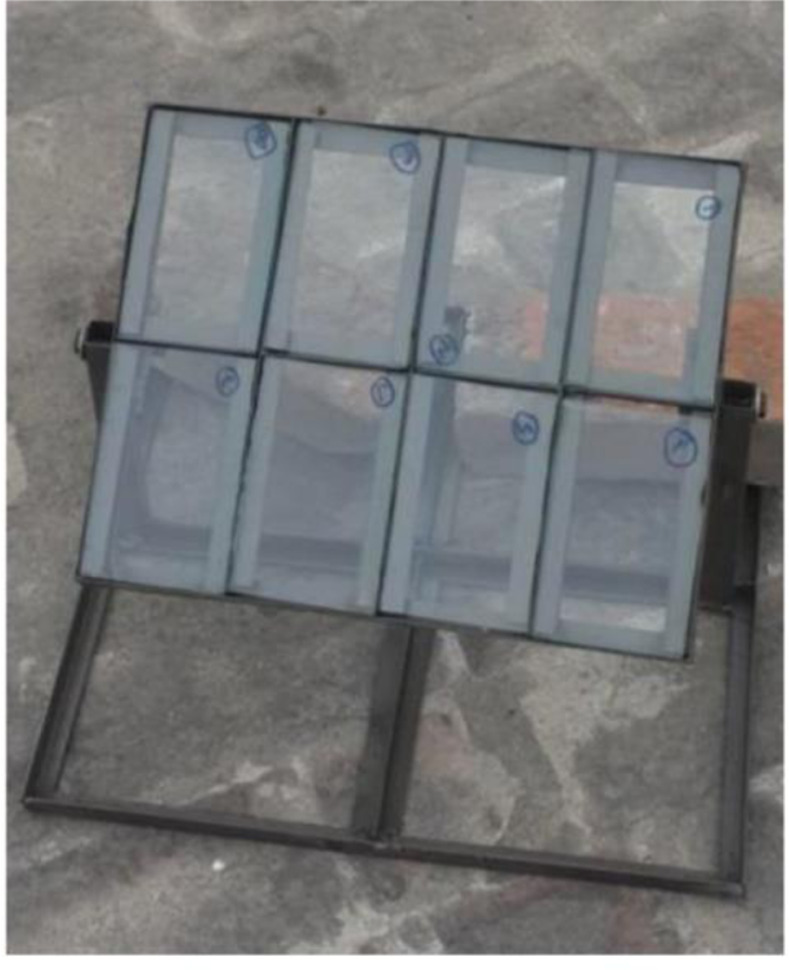
Dusted measurement setup.


The dust accumulation on the surface of the PV module under consideration was calculated using a setup. Eight pieces of glass slab are used in the frame as part of the dust measuring setup, as shown in Fig 5. Dust accumulation was quantified by placing glass slabs parallel to the panels in a specially designed frame at the same tilt angle of 33^o^. Initially, the glass slab’s weight was determined without any dust accumulation and recorded. After every week, the glass slabs are removed and weighed on a digital physical balance to measure the dust accumulation on each glass surface. Weighing the slabs before and after exposure each week allowed the calculation of dust density using a standard formula.

$$\text{Dust}\;\text{Density}=\frac{\text{Dust}}{\text{Area}\;\text{of}\;\text{Mirror}}$$

The novel application of a wiper-assisted cleaning mechanism incorporated a unique design featuring timed wiping cycles driven by a integrated motor and screw drive system. Likewise, the study introduces an original use of TiO_2_ nanoparticle coating to impart self-cleaning properties, providing the first evaluation of its maintenance-free benefits for solar panel maintenance under field conditions.

## 3. Results and discussion

The weekly recordings over the 5-week study period were analyzed to understand better how the key parameters changed over time for each PV panel setup when exposed to the same environmental conditions. The solar PV analyzer has been used to record and experimentally monitor the impact of dust on the V_oc_, I_sc_ and P_max_ of PV panels.

### 3.1. Electrical characteristics

**Fig 6:**
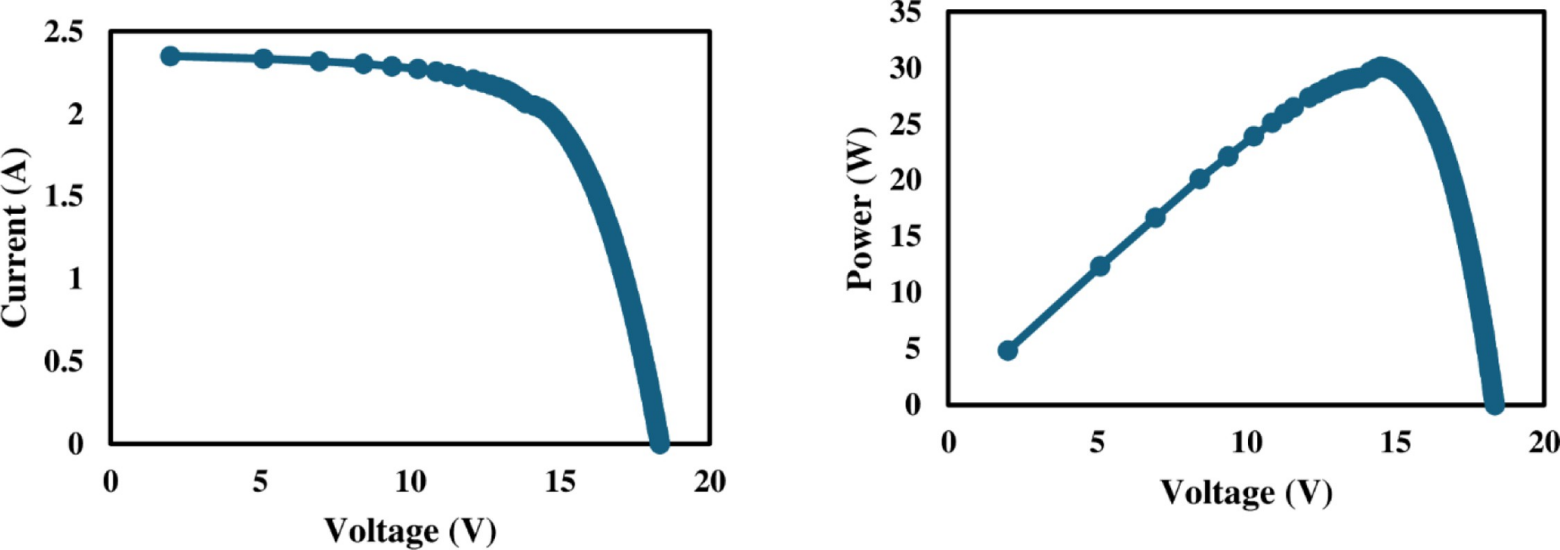
Electrical characteristics of reference solar PV panel.

**Fig 7:**
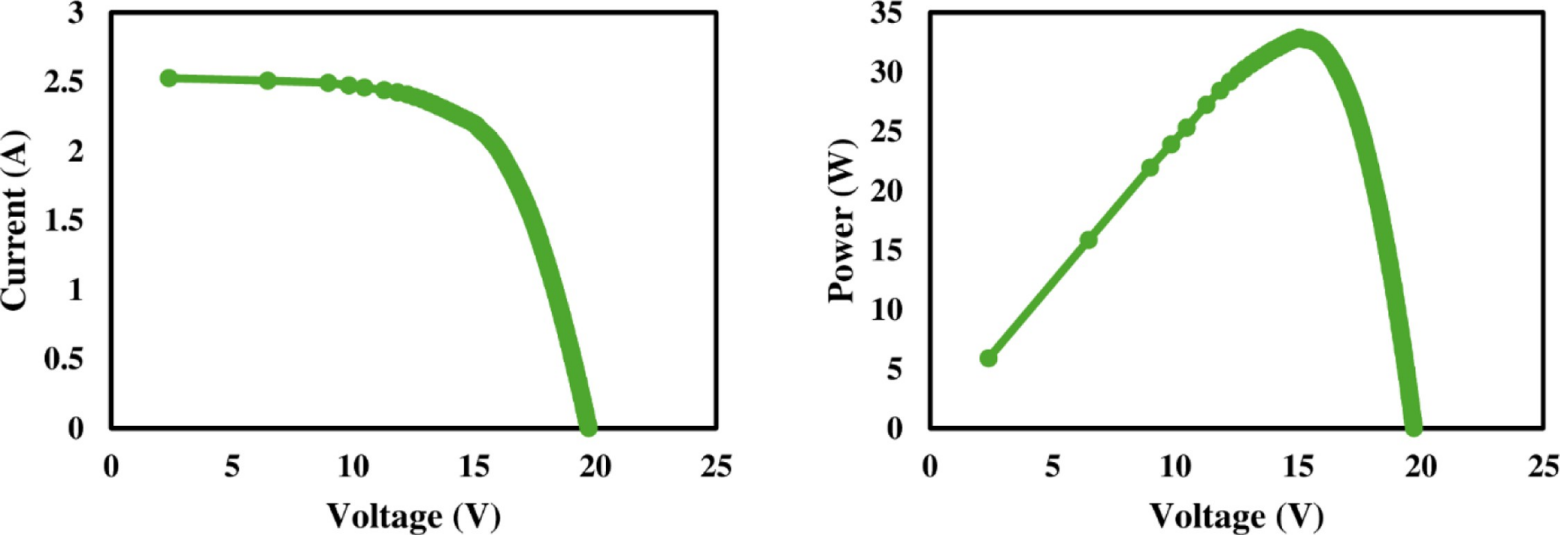
Electrical characteristics of nanocoated solar PV panel.

**Fig 8:**
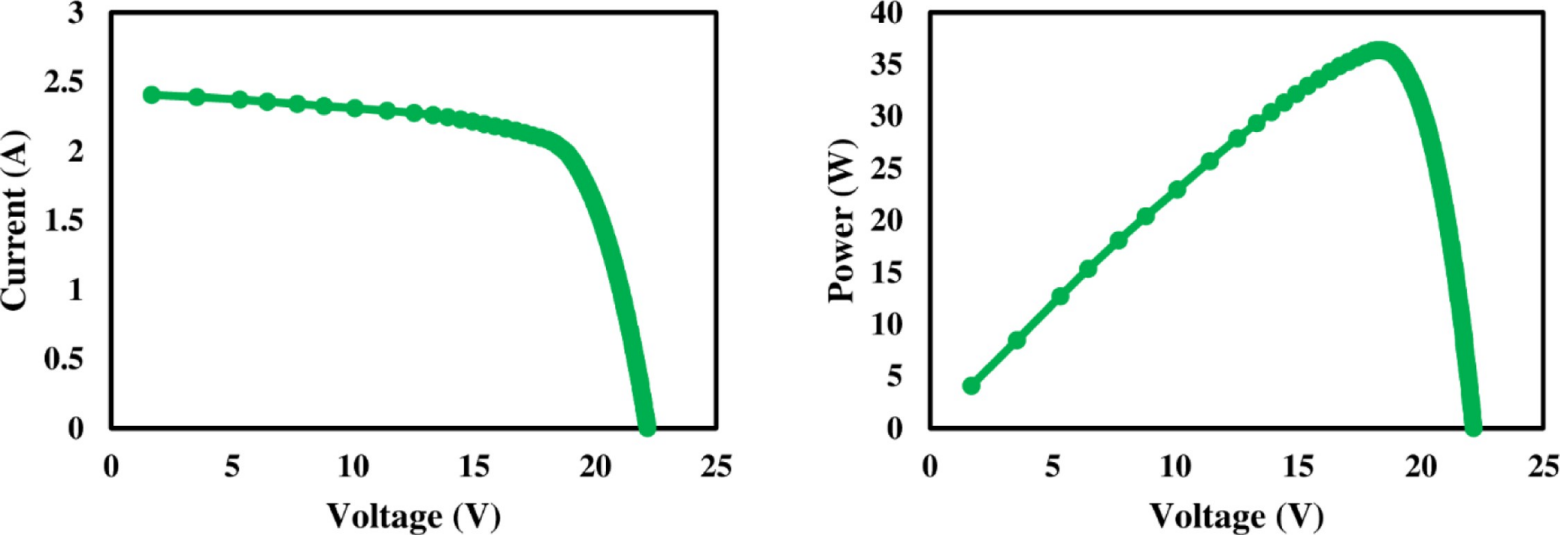
Electrical characteristics of wiper cleaning solar PV panel.

The I-V and P-V profiles of reference, nanocoated and SCW mechanism solar PV panels are given in Figs 6–8 respectively. The I-V and P-V curves provided a performance profile of each panel under varying light intensity levels. As expected, more dust accumulation corresponded to decreased I_sc_ values as dust blocked sunlight from reaching the cells.

**Fig 9:**
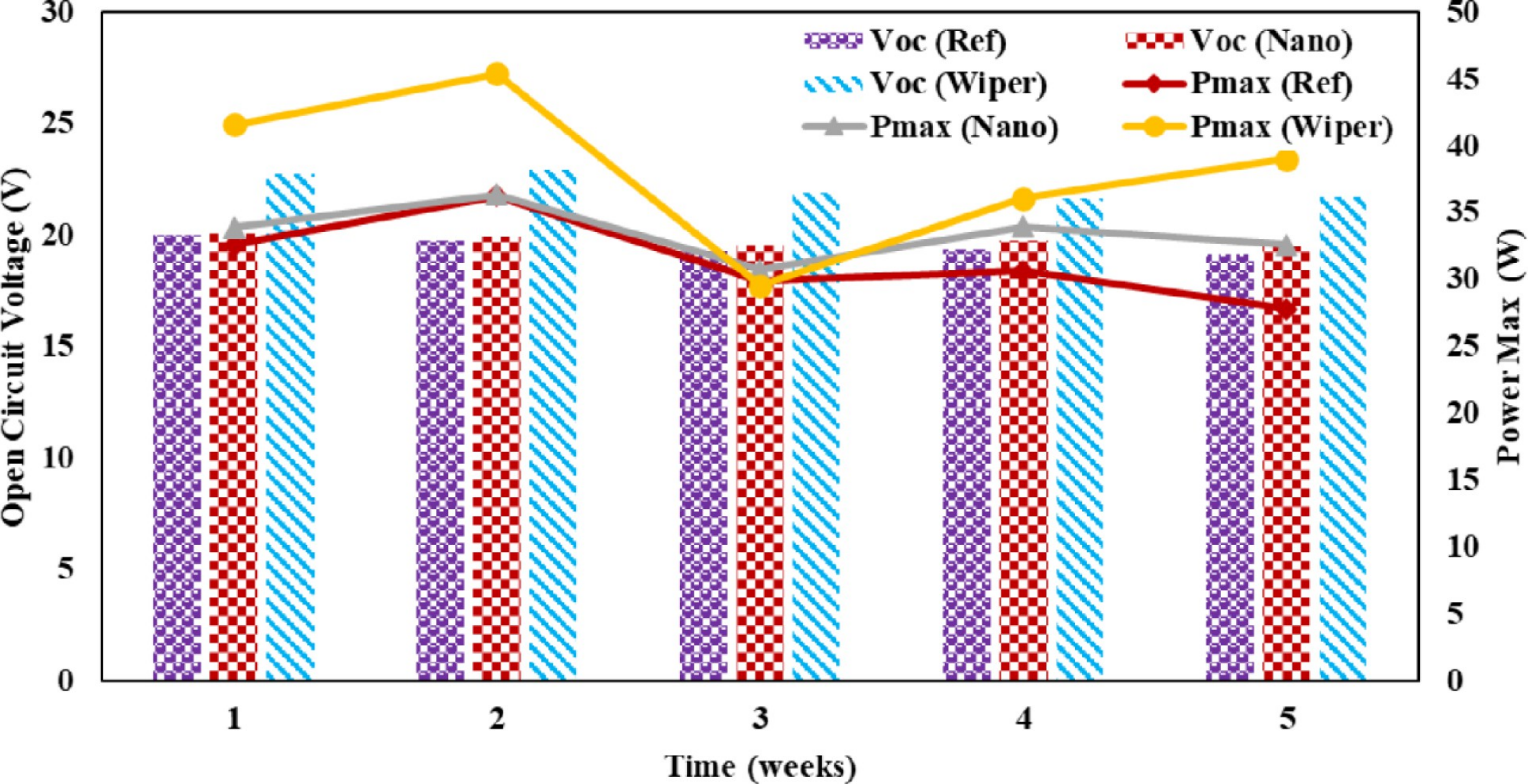
Open circuit voltage and maximum power of PV panels.

**Fig 10:**
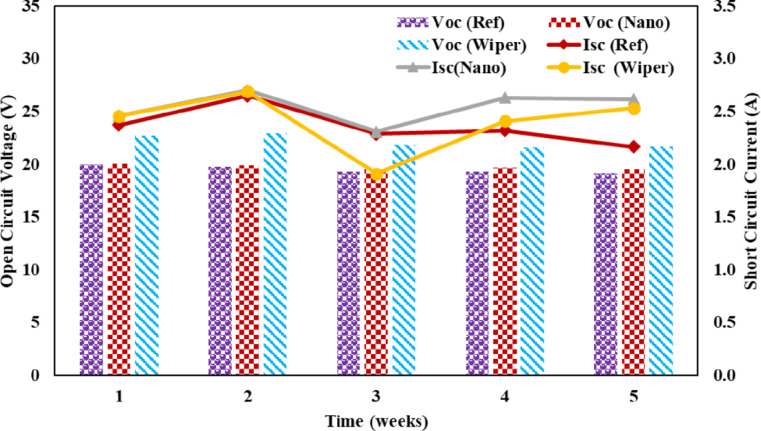
Short circuit and open circuit voltage of PV panels.

PV module electrical behavior solely depends on the intensity of incident radiance [37]. The P-V and I-V curves of solar panels determine their electrical characteristics under various solar radiation intensities. The amount of dust deposited has a considerable impact on the I_sc_. Due to dust buildup, the I_sc_ decreased in all three panels. It is clear from the Fig 9 that the low intensity of solar radiation in week three was because of cloudy weather. The maximum power output of PV panels eventually decreased because of this tendency, as seen in Fig 9. As illustrated in Fig 10, it can be observed that dust collection has no significant effect on the open circuit voltage. From the beginning of the research until its conclusion, there is a slight shift in the value of V_oc_. However, the operational temperature increase resulted in a considerable drop in the V_oc_ of all three panels, and this is consistent with research by Rao et al. [38].

The wiper mechanism panel’s Pmax was higher than the other panels at the beginning of the first week. Due to the hydrophobic properties of the coating, the nano-coated panel had a rising trend in power output in week two compared to the reference and wiper mechanism panels. The cloudy weather that followed the week decreased irradiation, resulting in reduced electricity output. Due to a clear sky, a power output rise was seen after week 4. The power output of both reference and nanocoated solar panels is reduced in week five due to dust buildup and dirty panel surfaces.

**Fig 11:**
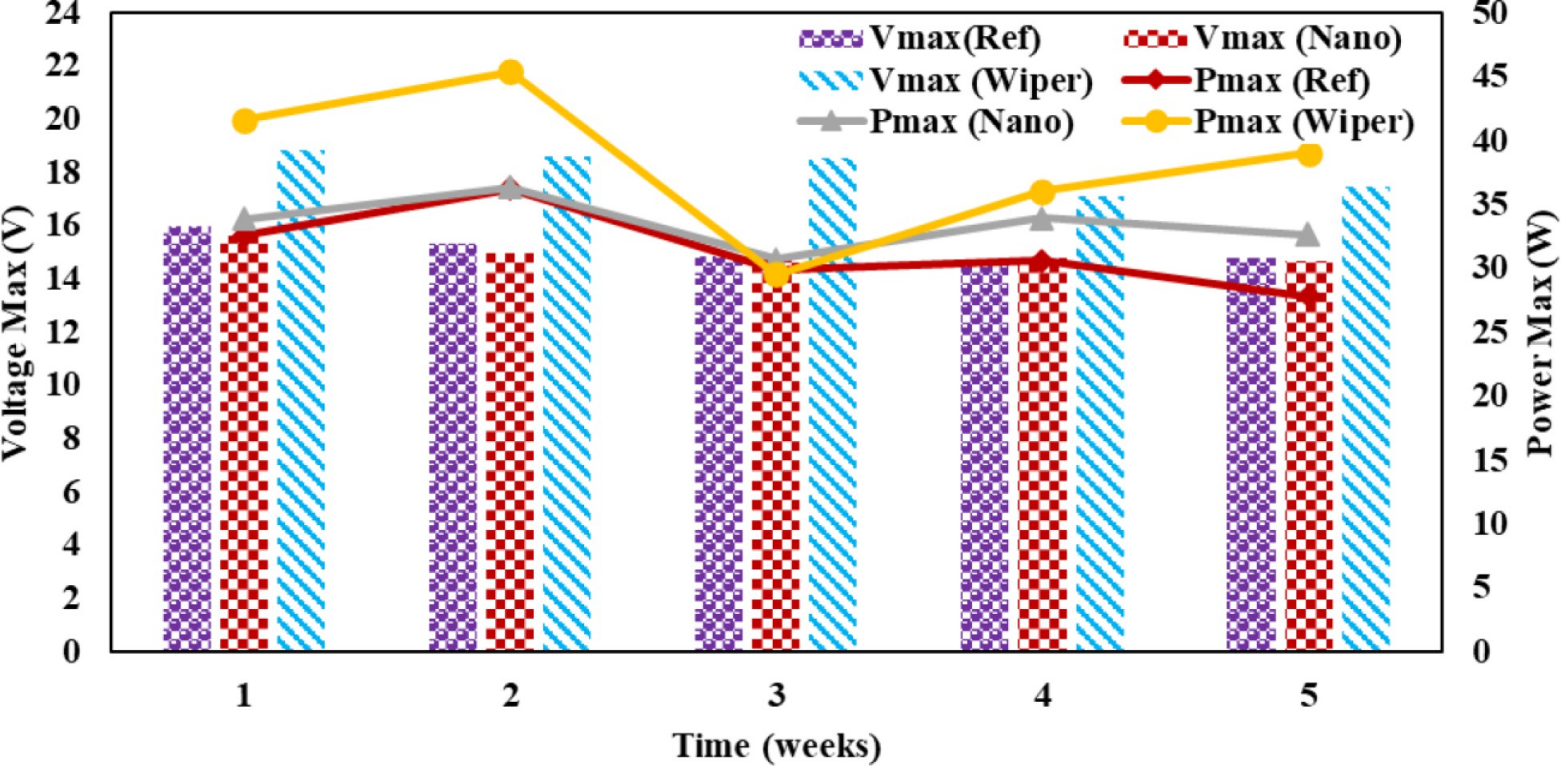
Maximum voltage and maximum power solar PV panels.

Fig 11 illustrates the behavior of maximum power production over five weeks. By the end of the study, the reference panel’s maximum drop in power output was 37%. While solar panels with cleaning mechanisms display a maximum power output drop of 23%, and nano-coated panels typically drop by 33%. Since the primary goal of the study was to determine how collected dust affected the performance of the PV modules, measurements for maximum power were made at midday on clear days when radiation intensity was at least 800 W/m^2^. This radiation intensity level meets the maximum intensity defined by ASTM E1036-85 and IEC/EN60891 standards [35].

**Fig 12:**
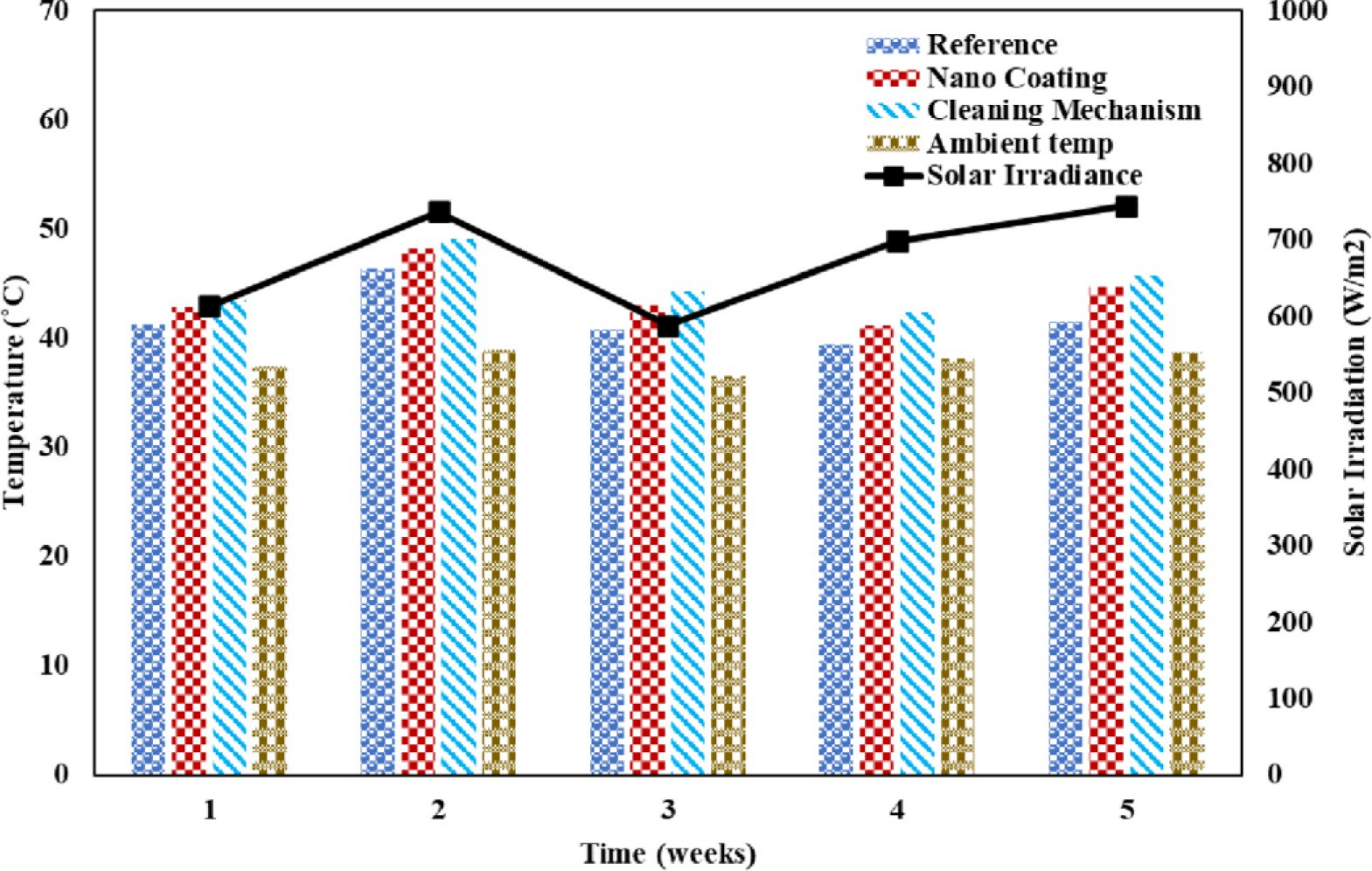
Back side and ambient temperature of PV panels along with solar irradiance.

Higher surface temperatures increase electrical resistance and lower efficiency. The reference panel likely recorded the coolest temperatures due to an insulating dust layer. Frictional heating from mechanical cleaning contributed extra heat to the SCW setup. Ambient temperature swings and variable sunlight followed anticipated patterns, highlighting their interlinked effects on panel performance through thermal impacts. Proper ventilation can mitigate some temperature losses. The comparison of the modules’ weekly backside and ambient temperatures and solar irradiance is shown in Fig 12. The relationship between module and ambient temperatures is direct. The rate of growth varies depending on the wind speed, though. In most cases, the sun irradiance rises from 9 AM (430 W/m^2^) to 12 PM (800 W/m^2^), is nearly constant until 2 PM, and then begins to fall. The cloudy weather in the third week’s was the cause of the drop in average solar irradiation. The temperature increase is due to dust buildup on the PV panel. Similarly, Chikate et al. conducted a research study highlighting the impact of temperature and irradiance fluctuations on various parameters of solar cells. These parameters play a crucial role in determining the performance of solar cells and modules under different conditions. The efficiency of a solar module is closely linked to these parameters, and any alterations in them can significantly affect the efficiency of the module [39]. The photovoltaic (PV) panel’s cell temperature typically increases as the ambient temperature rises. Only 20% of the energy received by a PV panel is generally transformed into useable energy, such as electricity; the remaining 80% is converted into heat energy. Both long-term and short-term losses are brought on by this heat energy. Power production and efficiency losses are examples of short-term losses. Long-term losses are what lead to a decline in performance.

In comparison to other panels, the reference panel’s surface temperature was the lowest over the whole research period. The temperature is increased due to the hygroscopic layer in the nano-coated panel. Due to extra heat generated by friction from cleaning operation and component latent heat, the PV panel with a wiper mechanism had the greatest surface temperature. Notably, results showed the nano-coated panels exhibited unexpectedly higher power output than the wiper system in the second week, highlighting original insights into their relative performance advantages.

### 3.2. Analysis of dust measurements

**Fig 13:**
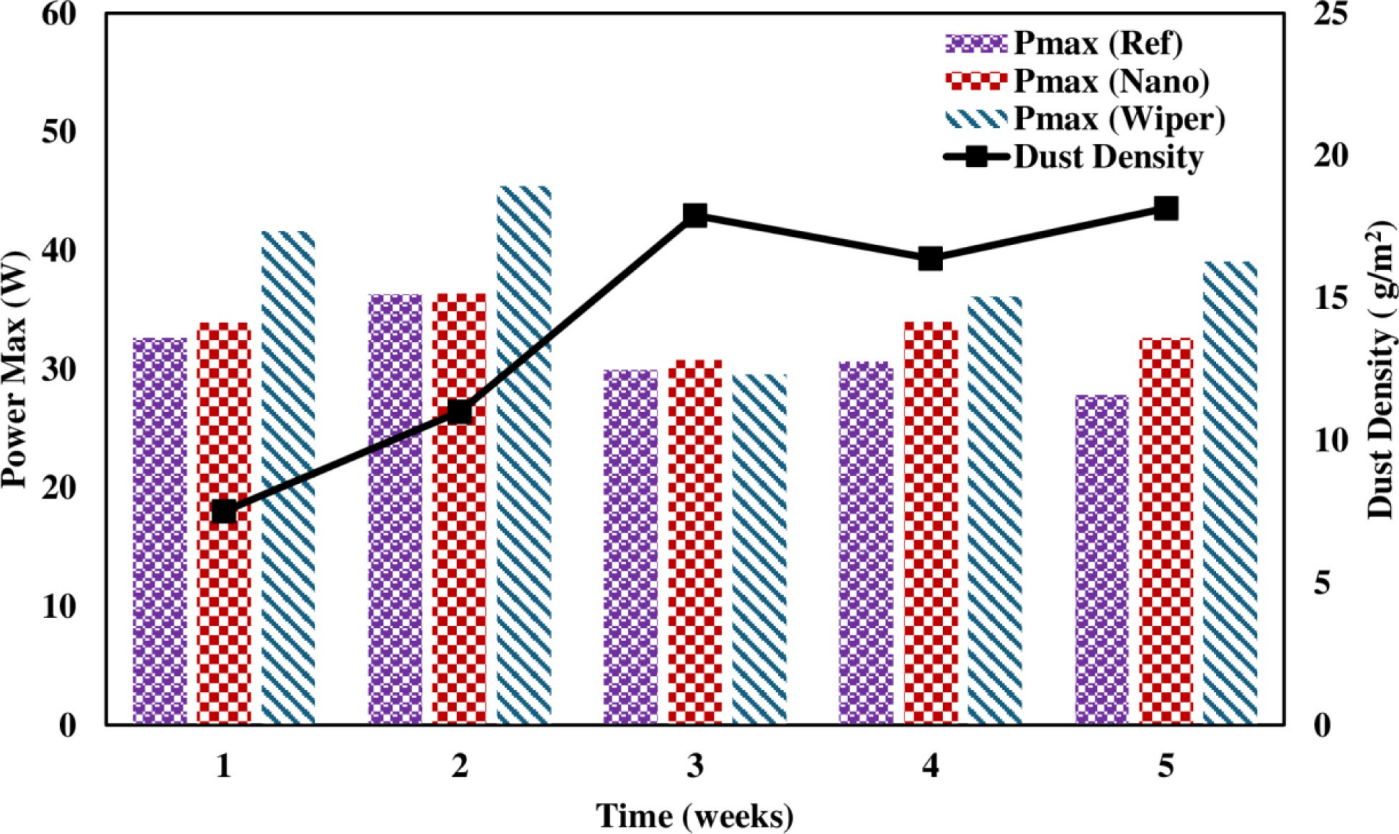
Pmax vs dust density.


The amount of collected dust on the surface was measured to determine the relationship between panel performance and dust deposition density. For this, various techniques were used, including weighing the sample before and after exposure [33], and measuring the weight of mirrors parallel to the panel’s surface after exposure to dust [32]. It was observed that the density of the dust accumulation increased with exposure time. The graph clearly shows the natural cleansing effects of cloudy weather and wind speed, which reduce the thickness of dust accumulation. [35]. As seen in Fig 13, the density of dust accumulation increases first, then reduces because of cloudy weather, and then increases again. This phenomenon can explain for specific high amounts of deposition in the first week. Some of the high estimates of dust deposition density are attributable to bird droppings in the dust slide. The solar intensity is another parameter to consider. It has been discovered that the loss in power output is substantially more severe for lower or greater sun intensities. This behavior is most likely due to the considerably increased light reflection impact of the accumulated dust [35].

$$\text{Panel}\;\text{Area}=\left(21.3*27*2.54\right)=1460.75\;{\text{cm}}^{2}$$

$$\text{Mirror}\;\text{Area}=\left(14*8.5\right)=119\;{\text{cm}}^{2}$$

Fig 13 shows a correlation between dust density and P_max_. PV panel power output is inversely related to dust density. As the dust density increases, power output decreases. Power output reduces as dust density increases. These results are in agreement with [40]. Semaoui et al. remarked that the accumulation of dirt and debris on the surface of solar panels can significantly reduce the short circuit current and overall power production of a photovoltaic (PV) system. As dust collects on the top of panels, natural cleaning methods such as rainfall and high-speed wind tend to increase power production. This increase in power output is attributed to a reduction in dust density on the Photovoltaic panel. Even after self-cleaning of PV panels, the power production occasionally stays low or declines. This is due to the PV panels receiving insufficient irradiation because of a partially or completely overcast sky. The photovoltaic panel’s power output is highly dependent on the availability of sunshine.

## 4. Cost benefit analysis

### 4.1. Return on investment of nano coated panel

The following estimates are made for the ROI of a panel with a nano-coating:

Cost of Nano coating for eight panels = Rs. 3000
Cost of Nano coating for one panel = Rs. 3000/8
  = Rs. 375
Nano coating weekly power savings = 4W
Nano coating’s annual power savings = 4*8*365/1000
  = 11.68 kWh
Electricity price per kWh = Rs. 30
Electricity price for 11.68 kWh = 11.68*30 = Rs. 350.4
ROI = Cost/yearly saving
  = 375/350.4
  = 1.0702 years

The estimates show that the nano coating has a ROI of 1.07 years.

### 4.2. Return on investment of SCW mechanism

The following estimates are made for the ROI of a panel with SCW mechanism:

SCW mechanism cost = Rs. 2200

The self-cleaning mechanism’s motor operates for 2 minutes daily, consuming 0.67 Wh of energy. Over a year, this amounts to 0.243 kWh, resulting in an annual electricity cost of Rs. 7.3 at Rs. 30 per kWh.

Weekly Power saving by self-cleaning mechanism = 9W
SCW mechanism yearly electricity saving = 9*8*365/1000 = 26.28 kWh
Electricity price per kWh = Rs. 30
Electricity price for 26.28 kWh = 30*26.28 = Rs. 788.4
ROI = Cost/yearly saving
  = 2207.3/788.4
  = 2.8 years

According to estimates, the SCW mechanism has a 2.8-year ROI.

With a payback period of 1.07 years for nanocoating compared to 2.8 years for the self-cleaning wiper system, nanocoating emerges as the more financially favorable option. Its lack of maintenance costs and lower initial investment make it an economically viable choice for solar panel cleaning. Table 4 presents the comparison of power losses of current study with published literature.

**Table 4:** Comparison of power losses of the current study with literature.

Reference	Power Loss
Present study	23–37% power loss, when dust density increased from 7.5 to 18.15 g/m^2^
Juaidi et al. [41]	9.99%
Zhao et al. [42]	7.58%
Aman et al. [43]	4.7% thin layer of dust. 10.17% thick layer of dust
Benghanem et al. [44]	28% due to dust accumulation
Dida et al. [45]	Dust accumulation caused 8.41% drop in maximum power output.
	Sandstorm led to 32% reduction in generated energy.
Abdelsalam et al. [46]	Power losses linearly related to dust density, 1.27% per g/m^2^.
	Absolute reduction in output power increased by 8.46% after 41 days.

## 5. Conclusion

The current study focuses on a detailed comparative performance analysis of two distinct self-cleaning mechanisms: self-cleaning wiper (SCW) and nano-coating method on solar panels subjected to standard atmospheric conditions in Pakistan. The primary objective was to evaluate the effectiveness of these mechanisms in mitigating the adverse effects of dust accumulation on solar panels. This study examined the performance of three 50 W PV panels over a five-week period, focusing on the impact of dust accumulation and the effectiveness of different cleaning mechanisms. Dust measurements were conducted using glass slabs, and a solar PV analyzer (Prova 200A) was employed to assess panel efficiency. The results revealed that the open circuit voltage V_oc_ remained unaffected by dust buildup, maintaining consistent levels despite dust accumulation [37]. Whereas, short circuit current I_sc_ showed is significant decrease with increased dust deposition. Due to an increase in dust density from 7.5 to 18.15 (g/m^2^), the percentile decreases in power output of the SCW mechanism, nanocoated panel, and reference panel are about 23%, 33% and 37%, respectively. By comparing the PV performance of the cleaning wiper mechanism and nanocoated panels with reference panel it was concluded that the SCW method for solar panels is crucial for maintaining the optimal efficiency in dust-prone areas. Moreover, due to the hydrophilic nature of the nano-coating, it repels water droplets and dust particles, resulting in lower power losses compared to the reference panel. This pioneering study therefore offers novel guidelines on the feasibility of these two innovative self-cleaning techniques as low-cost, sustainable solutions optimized for Pakistan’s atmospheric conditions through the first direct side-by-side evaluation.

## Supporting information

S1 Data (XLSX)

## Abbreviations

SCW: Self-Cleaning Wiper Mechanism
PV: Photovoltaic
LDR: Light Dependent resister
V_oc_: Open Circuit Voltage
I_sc_: Short Circuit Current
P_max_: Maximum Power Output
