# Correction: Evaluation of self-cleaning mechanisms for improving performance of roof-mounted solar PV panels: A comparative study

**DOI:** 10.1371/journal.pone.0313662

**Published:** 2024-11-07

**Authors:** Danish Hameed, Allah Ditta, Muhammad Wasif Bajwa, Sibghat Ullah, M. A. Mujtaba, Yasser Fouad, M. A. Kalam, Manzoore Elahi M. Soudagar

In the Funding statement, the Supporting Project Number from the funder, King Saud University, Riyadh, Saudi Arabia, is listed incorrectly. The correct Supporting Project Number is: RSPD2025R698.
